# *Toxocara vitulorum* cuticle glycoproteins in the diagnosis of calves’ toxocariasis

**DOI:** 10.14202/vetworld.2019.288-294

**Published:** 2019-02-20

**Authors:** Eman E. El Shanawany, Soad E. Hassan, Adel A.- H. Abdel-Rahman, Eman H. Abdel-Rahman

**Affiliations:** 1 Department of Parasitology and Animal Diseases, National Research Centre, Giza, Egypt; 2 Department of Chemistry, Faculty of Science, Menoufia University, Shebin-El Kom, Egypt

**Keywords:** affinity chromatography, calves toxocariasis, Con A, enzyme-linked immunosorbent assay, mass spectrometric analysis, N-acetylglucosamine

## Abstract

**Aim:**

The current study was designed to isolate and characterize *Toxocara vitulorum* glycoprotein antigens and then to evaluate its potency in accurate diagnosis of toxocariasis.

**Materials and Methods:**

*T. vitulorum* glycoprotein fractions were isolated using Con-A affinity chromatography. The fractions characterized using sodium dodecyl sulfate-polyacrylamide gel electrophoresis (SDS-PAGE), and immunoblot assay. Mass spectrometric analysis was used for identification of proposed structure of the N-acetylglucosamine (GlcNAc) fraction. Enzyme-linked immunosorbent assay (ELISA) was used to assess the diagnostic potential of the isolated fractions.

**Results:**

Surface of *T. vitulorum* adult worm revealed two glycoprotein fractions rich in glucose (Glc) and GlcNAc. Three bands of molecular weight 212kDa, 107 kDa, and 93 kDa were detected in Glc fraction by SDS-PAGE. These bands were also detected in GlcNAc fraction with an additional band of 49 kDa. GlcNAc fraction showed more diagnostic potency of calves’ toxocariasis; 79% than Glc fraction; 46.9% by indirect ELISA. The additional band of 49 kDa in GlcNAc fraction is probably responsible for its higher diagnostic potentials. Western blotting verified the immunoreactivity of the Glc and GlcNAc isolated fraction as they reacted with calves sera infected with toxocariasis. The proposed structure of GlcNAc fraction was Ser-Meth-Arg-O-methylated GlcNAc.

**Conclusion:**

GlcNAc-rich fraction of *T. vitulorum* can be successfully utilized in the diagnosis of calves’ toxocariasis.

## Introduction

A large gastrointestinal nematode, *Toxocara vitulorum*, is widely spread in buffalo calves, and it causes an economic problem due to high mortality rate, anemia, diarrhea, weight loss, anorexia, and obstruction of the small intestine [1-4]. Calves could be infected with *T. vitulorum* larvae through colostrum and milk from infected mothers. The larvae mature in calves’ small intestines and shed eggs in feces. The alternative life cycle takes place by calves ingesting embryonated eggs from the environment. Larvae go through migration in liver, lung muscles, and brain. The prevalence of large ruminant’s toxocariasis in Egypt is considered high [2]. Approximately 47.9% of slaughtered cattle and buffaloes were infected in Dakahlia [2]. In western countries, the seroprevalence of toxocariasis was ranged from 2% to 5%. However, in tropical countries, *Toxocara* infection has been found to be higher, approximately 63% [5]. Toxocariasis control is not easy because the larvae migrate in the tissues, remaining as dormant or hypobiotic parasites.

Definitive diagnosis of toxocariasis is based on the basis of clinical signs, necropsy findings, fecal examination, and serological tests. However, the fecal examination is unsuccessful to detect infection at the prepatent period and in mild infections of calves. Hence, the serology is the more suitable alternative way for diagnosis. Serodiagnosis of toxocariasis is often performed using different antigens, such as excretory-secretory antigen of *T. vitulorum* infective larvae, perienteric fluid antigen, and crude antigen of adult *T. vitulorum* [6-8]. However, cross-reactivity and their weak ability to detect *T. vitulorum* are an obvious problem. Thus, the isolation of specific antigens with the highly sensitive and accurate diagnostic ability is an important issue [9,10]. Recently, the researcher’s attention was directed toward carbohydrate antigens. The glycoprotein structure was exhibited its ability to dominate host antibody response at different helminths [11,12]. Moreover, Długosz and Wiśniewski [10] proved that there is a wealth of evidence, suggesting that glycan helminths are involved in epitope formation, so it is considered as antigens for diagnostic purposes.

A large number of studies were interested in *Toxocara canis* and *Toxocara cati* glycoprotein antigens [13-15]. Meghji and Maizels [16] characterized the excretory-secretory product of *T. canis* larva which represents high content of N-acetylgalactosamine (GlcNAc) and galactose. Mass spectrometry of the O-linked sugars determined it to be two related trisaccharides, 2-O-Me-Fucα1-2(4-O-Me)Galβ1-3-GalNAc and 2-O-Me-Fucα1-2Galβ1-3GalNAc, differing only in whether the central galactose sugar has an O-methyl side chain [17]. Schabussova *et al*. [18] succeeded in chemical synthesize O-glycan of *T. canis* and proved its potency in the diagnosis of toxocariasis in human. In addition, Khoo *et al*. [19] proved that *T.canis* excretory-secretory (TES) antigen was N-linked glycan with Man2Man-GlcNAc-GlcNAc side chain. Accurate diagnosis is important for understanding the epidemiology of toxocariasis and establishing preventive measures. In addition, there is no available literature concerned *T. vitulorum* glycoprotein antigens and its ability for the diagnosis of animal’s toxocariasis.

Hence, the objective of the present study is to isolate and characterize *T. vitulorum* glycoprotein antigens. Furthermore, the use of this antigens in accurate diagnosis of calve’s toxocariasis was another target.

## Materials and Methods

### Ethical approval

Sample collection from animals were reviewed according to Egyptian governmental regulations. Serum samples and adult *T. vitulorum* worms were collected from buffalo calves slaughtered at the government abattoir El Moneeb, Giza, Egypt.

### Serum samples

Random serum samples were collected from 49 buffalo calves. Negative (n=8) and positive serum samples (n=10) were collected from calves and confirm its negativity and positivity using fecal examination. Serum samples were labeled in serial numbers and stored at −20°C until use.

### Parasite

Worms were washed thoroughly by de-chlorinated water; worm cuticle was separated, collected, and frozen until use.

### Preparation of *T. vitulorum* cuticle antigen

*T. vitulorum* cuticle was homogenized in phosphate-buffer saline (PBS) at pH 7.2. The cuticle extract was centrifuged at 13,000 rpm for 30 min at 4°C and supernatant was aliquoted then preserved at −20 until use. The protein concentration of extract was estimated by the method of Lowry *et al*. [20].

### Isolation of *T. vitulorum* cuticle glycoprotein antigens

Prefilled Concanavalin ensiformis (Con A) column was obtained from Sigma Chem. Co. St. Louis, USA, and then it was utilized for the isolation of glycoprotein fractions of *T. vitulorum* cuticle antigen as described by Abdel-Rahman *et al*. [21]. Briefly, *T. vitulorum* cuticle antigen was applied to the column and allowed to mix overnight at 4°C. The column was washed with 0.01M PBS pH 7.3 until the unbound part of the antigen was passed completely from the gel. The bound glycoproteins were eluted with 50 mM glucose (Glc) to obtain the first fraction and then eluted with 50 mM N-acetyl glucosamine for obtaining a second fraction (GlcNAc). The fractions were checked for protein content by the method of Lowry *et al*. [20].

### Sodium dodecyl sulfate-polyacrylamide gel electrophoresis (SDS-PAGE)

The technique was performed according to the procedures described by Laemmli [22] for the characterization of Glc and GlcNAc fractions. The gel stained with silver stain according to the method of Wray *et al*. [23]. Molecular weight standards (14-220 kDa) were electrophoresed in the same gel for comparative purposes.

### Western blotting

The resolved proteins were transferred to 0.45-μm nitrocellulose membranes (Serva Electrophoresis, Germany). Membranes were subsequently blocked with 5% skimmed milk in Tris-buffer saline with Tween^®^ 20 (TBST) buffer consisting of Tris, 125 mM NaCl, and 0.1% Tween 20 for 1 h at room temperature followed by incubation with primary antibodies against *T. vitulorum* (control positive serum sample) diluted at 1:100, overnight at 4°C. Excess antibodies were removed by extensive washing in TBST, and blots were then reprobed with horseradish peroxidase-conjugated anti-bovine IgG (1/1000 dilution, Abcam). Membranes were then extensively washed with TBST and treated with enhanced chemiluminescence reagent (Optiblot™, Abcam, UK). Light-sensitive films (Fuji, Japan) were used for X-ray film exposure [24].

### Chemical elucidation of GlcNAc glycoprotein fraction structure

GlcNA homogenous fraction was applied on thin-layer chromatography (TLC) EM silica gel 60 F sheet (0.2 mm). Use CHCl3/CH3OH (9:1) as the developing eluent with rate flow (Rf)=0.2. U.V model UVGL-58 was used to detect the spot. Varian MAT 311A spectrometer was used for yield data of mass spectra.

### Enzyme-linked immunosorbent assay (ELISA)

The assay was utilized to evaluate the diagnostic potential of the two isolated fractions Glc and GlcNAc in the diagnosis of toxocariasis in buffalo calves. The test was performed according to Oldham [25]. The optimal concentration of the antigens and dilution of serum was determined using checkerboard titration.

Briefly, the plate was coated with isolated fractions, either Glc or GlcNAc, in carbonate buffer (100 µl/well) and incubated overnight at 4°C. After washing, the unbinding sites were blocked with 0.1M bovine serum albumin in carbonate buffer and incubated for 1 h at room temperature. The plate was washed repeatedly, and serum samples in dilution (1:100) were added (100 µl/well) and incubated for 90 min at 37°C. After washing, the conjugate was added (anti-bovine IgG horseradish peroxidase which was purchased from Sigma Chem. Co. St. Louis) to each well and the plate was incubated for 1 h at 37°C. Then, the substrate was added, Ortho-phenylenediamine and the plate was read spectrophotometrically at 405 nm. The cutoff values were calculated according to Almazan *et al*. [26].

### Sensitivity and specificity

The following definitions were used to calculate the corresponding diagnostic parameters: True-positive (TP) values, sera from calves with toxocariasis showing positive readings; false-negative (FN) values, sera from calves without infection of toxocariasis showing negative readings; false-positive values (FP), sera from healthy calves without toxocariasis showing positive readings; true-negative values (TN), sera from healthy calves showing negative readings; specificity=(TN/FP+TN)×100; and sensitivity=(TP/TP+FN)×100 [27].

### Statistical analysis

Data were analyzed for the means and standard deviation. Significance of the results was evaluated using one-way ANOVA computer programs.

## Results

### Diagnostic performance of the two isolated glycoproteins

**Figure-1:**
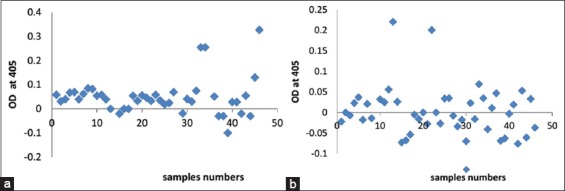
(a) Scattered graph represents diagnostic potential of *Toxocara vitulorum* cuticle N-acetyl glucosamine isolated fraction. (b) Scattered graph represents diagnostic potential of *T. vitulorum* cuticle glucose isolated fraction.

The two glycoprotein isolated fractions exhibited high diagnostic sensitivity where the GlcNAc fraction showed 80% compared with 60% for Glc fraction. The two glycoproteins showed important differences in their diagnostic specificity. Where the GlcNAc showed 75% specificity compared with 50% for Glc fraction with significant difference (p>0.01) as shown in (Table-1). GlcNAc fraction had an excellent ability to discriminate between seropositive and seronegative calve’s serum samples where 10 samples were negative in GlcNAc fraction with infection of 79% (Figure-1a) compared with 26 samples which were negative in Glc fraction with infection of 46.9% (Figure-1b). The cutoff value was 0.25.

**Table-1:** Specificity and sensitivity of isolated *T. vitulorum* glycoproteins for diagnosis of calves’ toxocariasis.

Tested fractions	n	Disease statues				Diagnostic sensitivities and specificities	
	
		TP	FN	FP	TN	Sensitivity (%)	Specificity (%)
GlcNAc fraction	Positive=10	8	2	2	6	80	75
Glucose fraction	Negative=8	6	4	4	4	60	50

Sensitivity= (TP/TP+FN) 100×, Specificity= (TN/FP+TN) 100×; TP=True positive, FN=False-negative, FP=Flase-positive, TN=True-negative. Cutoff value of=0.25, *T. vitulorum=Toxocara vitulorum*

### Electrophoretic profile of two isolated fractions of *T. vitulorum*

**Figure-2:**
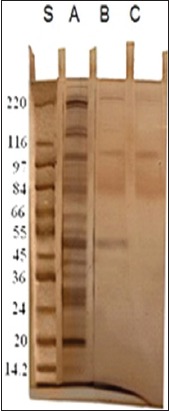
Sodium dodecyl sulfate-polyacrylamide gel electrophoresis analysis of cuticle antigens of adult *Toxocara vitulorum* lane A, N-acetyl glucosamine (GlcNAc) isolated fraction lane B and glucose isolated fraction (Glc)lane C. Lane S: protein molecular weight marker.

The electrophoretic pattern of cuticle crude antigen showed 20 bands of different molecular weights ranged from 232.1 to 14.7 kDa. Glc fraction yielded three bands of molecular weights 212, 107, and 93 kDa, while GlcNAc fraction consisted of four bands at molecular weights 212, 107, 93, and 49 kDa (Figure-2).

### Immunogenic bands of two isolated glycoprotein fractions

**Figure-3:**
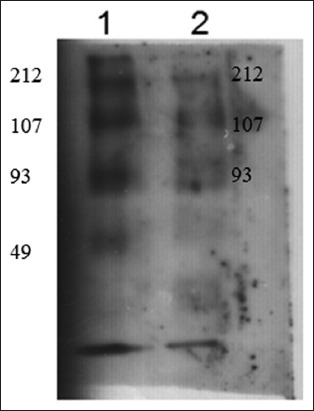
Western blotting showing immunoreactive bands of *Toxocara vitulorum* cuticle two isolated fractions. Lane 1: N-acetyl glucose amine fraction (GlcNAc). Lane 2: Glucose fraction (Glc).

All bands of the two isolated fractions were reacted with naturally infected calves’ sera. Glc fraction exhibited three bands of molecular weight 212, 107, and 93kDa, while four bands of molecular weight 212, 107, 94, and 49 kDa were detected in GlcNAc fraction (Figure-3).

### Chemical structure of immunogenic isolated fraction

One spot was getting on the TLC when using MeOH/CHCl_3_ (80:20 v/v) as an eluent. By immersing the TLC sheet in 5% H_2_SO_4_ and heating, it gives burning red color indicates the presence of GlcNAc sugar moiety. The proposed structure of GlcNAc isolated fraction was elucidated using mass spectrum which observes different peaks. The peak at m/z=491 is a sign for the M^+^ - serine (Ser) residue. The fragmentation at m/z=422 was proven the presence of M^+^ - arginine (Arg) residue. The M^+^ - methionine (Meth) residues observe through the presence of a peak at m/z=447. The peak at m/z=424 is a marker for the GlcNAc-GlcNAc residue [M]. The electrophoresis gives overall molecular weight at 460 kDa which mean that the glycopeptide polymer is:


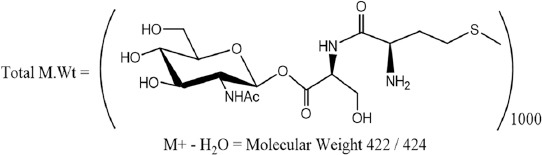


**Figure-4:**
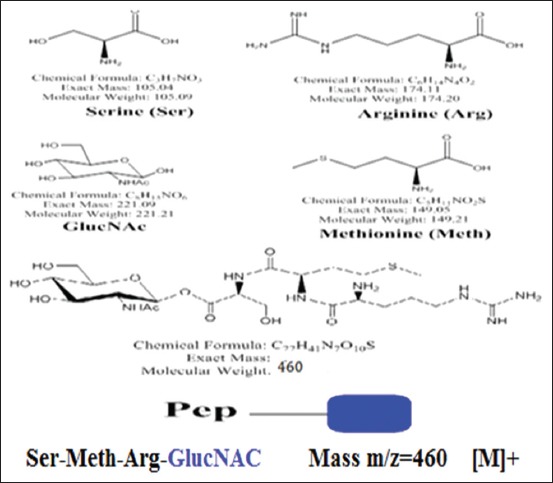
Proposed structures of O-glycans from isolated GlcNAc glycoprotein fraction; using mass spectrometry.

The monomer name is:

(S)-((2S,3R,4R,5S,6R)-3-acetamido-4, 5-dihydroxy-6-(hydroxymethyl)tetrahydro-2H-pyran -2-yl)2-((R)-2-((S)-2-amino-5-guanidinopentanamido)-4-(methylthio)butanamido)-3-hydroxypropanoate (Figure-4).

## Discussion

The role of parasite glycan is important in determining antigenicity and has been shown to be involved in parasite survival, infectivity, and host-cell recognition [28,29]. *Toxocara* glycan components have remarkable biological properties [30]. Although *T. canis* and *T. cati* had intensive research for their glycoproteins structural characterization, *T. vitulorum* faced lack of study about their glycoproteins. Hence, in the current study, we focused a spotlight on *T. vitulorum* cuticle glycoproteins.

Two glycoprotein fractions, Glc and GlcNAc, were isolated from *T. vitulorum* cuticle by lectin affinity chromatography. By SDS-PAGE, Glc fraction was resolved to three bands of molecular weights 212, 107, and 93 kDa compared with four bands of molecular weight 212, 107, 93, and 49 kDa inGlcNAc fraction. Using immunoblotting assay, all these bands were reacted with calves naturally infected sera. In another study, four bands of different molecular weights (32, 55, 70, and 120 kDa) were detected in glycoprotein antigen of *T. canis* larval stage [31]. This difference in isolated glycoprotein molecular weight may be related to the difference in the *Toxocara* species. However, Rubinsky-Elefant *et al.*, [32] found that *T.canis* have diagnostic markers with low molecular weights (29-38 and 49-54 kDa). This result proves our finding that GlcNAC fraction which has low molecular weights (49 kDa) was more sensitive and specific in the diagnosis of calves toxocariasis. In the current study, mass spectrometry was used for further structural characterization of the most isolated diagnostic fraction (GlcNAC) cuticle glycoprotein of *T. vitulorum*. The proposed structure was Ser-Meth-Arg-O- methylated GlcNAc. High level of GlcNAc as a major component of the total glycan pool has been described for other helminths. However, some common structures reported in *T. canis* and *T. cati*, such as the GalNAc, galactose, mannose, and multifucosylated termini [17-19], were not found in the currently isolated glycoprotein fraction. The interpretation of this notice confirms that at the level of structural characterization, there is a common compound between *Toxocara* species as GlcNAc, but this structure is not necessarily occurred in all *Toxocara* species. It proved existence of unique structure of *T. vitulorum* (Ser-Meth-Arg-O-methylated GlcNAc) not necessarily exists in other helminths. The isolated glycoprotein here is O-linked trisaccharide structures, and this proposition is consistent with their being the most abundant O-glycans in other glycoprotein isolated from *T. canis* and *T. cati* [17,19]. Khoo *et al*. [19] isolated O-methylated GalNAc from *T. canis* larval stage ES glycoprotein antigen.

Furthermore, Khoo *et al*. [17] isolated other glycoprotein fraction from TES of *T. canis* which is also O-glycan galactose and N- acetyl galactoseamine rich. A possible interpretation of the present result is that the degrees of O-glycans in TES of *T. canis* are more abundant than N-glycosylated glycoproteins [17]. Furthermore, the methylation of the isolated GlcNAc fraction is confirmed by Schabussova *et al*. [18] who proved that Toxocara species has mono-methylated trisaccharide and dimethylated structure is shared epitope present in all *Toxocara* species.

To elucidate immunogenicity of isolated glycoprotein fractions, ELISA was used. IgG was used because it is considered the best marker which detected in all patients with visceral, ocular, and mixed forms of the disease Rubinsky-Elefant *et al*. [32]. The presented result reported diagnostic sensitivity for GlcNAc and Glc fractions was 80% and 60%, respectively, while specificity was 75% and 50%, respectively. The specificity and sensitivity were calculated according to Parikh *et al*. [27] where the specificity is the ability of a test to correctly classify an individual as disease-free and sensitivity is the ability of a test to correctly classify an individual as “diseased.” Furthermore, in testing random calves’ sera, it was observed that GlcNAc fraction was the most potent and showed 79% infection percentage compared with 46.9% with Glc fraction. It was clear that two glycoprotein fractions were potent in the diagnosis of calves’ toxocariasis with the observation that GlcNAc fraction was more sensitive and specific than Glc fraction. The diagnostic potency of other *Toxocara* species (*T. canis)* glycoprotein was previously studied by Schabussova *et al*. [18] who synthesized the O-methylated GlcNAcand proved the reactivity of synthetic forms of these glycans to parasite-specific antibodies. Furthermore, Khoo *et al*. [17] recognized O-methylated trisaccharides carbohydrate of TES *T. canis* which exhibits reactivity with monoclonal antibodies. Moreover, Koizumi *et al*. [9] proved the potency of *Toxocara* glycans in the diagnosis of human toxocariasis by recognizing serum IgG. The presented results are consistent with the study of Długosz and Wiśniewski [10] who showed that glycosylation of TES and recombinant mucins affect the binding of immunoglobulins IgG1. Deglycosylation of these antigens significantly decreased its recognition by IgG1 immunoglobulins. In general, the diagnostic potential of glycoprotein antigen related to its ability to enhancing processing, presentation, and recognition antigens by APCs and T/B-lymphocytes [33,34] which leading to T cell-dependent and relatively strong IgG1 responses [35,36]. The efficacy of GlcNAc fraction may be attributed to nature of this glycoprotein fraction and/or presence of a band of molecular weight 49 kDa which detected only in GlcNAc fraction. Furthermore, the possible interpretation is that the high specificity and sensitivity of GlcNAc glycoprotein isolated in the presented result is that *Toxocara* trisaccharides show similarity with human blood group antigens, especially H or O specificity of ABO system. This resemblance interpreted the high titers of isohemagglutinin in animals with toxocariasis; this is due to reactivity of ABO system with experimental antisera to *Toxocara* and related parasites of the *Ascaris* genus [37,38]. The ambiguity of seroreactivity with cuticle *Toxocara* also reflects the presence of unmodified blood group antigens in this material [39,40]. Hence, the O-methylated glycan GlcNAc isolated fraction may consider a diagnostic target, on the basis of our limited tests, which is not reactive with mammalian blood groups.

## Conclusion

We have isolated two glycoprotein fractions, Glc, and GlcNAc, from *T. vitulorum* cuticle to study their efficacy in the diagnosis of toxocariasis by ELISA. The results demonstrated that GlcNAc fraction may serve as a diagnostic tool to detect *T. vitulorum* infections in buffalo’s calves in large scales.

## Authors’ Contributions

EHA designed the paper. SEH and EEE: Serum samples and parasite collection. EEE, SEH, and EHA: Preparation of *T. vitulorum* cuticle antigen. EEE and SEH: Performed SDS-PAGE. EEE: Isolation of *T. vitulorum* cuticle glycoproteins antigen, carried out western blotting and ELISA, analysis and interpretation of the data and drafting of the manuscript. AAHA: Chemical elucidation of GlcNAc glycoprotein fraction structure. All authors revised, read and approved the final manuscript.
